# On the Mechanism or Nature of Atrophy

**Published:** 1845-01

**Authors:** 


*On the Mechanism or Nature of Atrophy.*—The following extract, from a standard work of medical literature on the continent, is a tolerably fair sample of the spirit of abstruse and fanciful speculation, if not of the philosophic *clairvoyance*, that characterises so many of the writings of the German physiologists.

"Every act of nutritive crystallisation takes place on the outside of the capillary vessels, in a fluid derived from the blood (*plasma*, vel *cysto-blastema.*) The element of formation is the cellule. This possesses a proper individual life, in virtue of which it is developed (plastic force;) moreover, it has the faculty of inducing peculiar chemical changes in the materials which it derives from the *cysto-blastema* by its inner and outer surfaces (metabolic force.) Atrophy may be caused, 1, either by the general nutritive fluid ceasing to circulate in the minute vessels, and by the consequent desiccation of the organ; 2, or from the *plasma* being deficient in the formative materials; 3, or from a morbid condition of the cellule itself, rendering it unfit to fulfil its functions; or 4, from some peculiarity in the state of the nervous influence. The cause of atrophy may also reside in the work of decomposition—which consists in the return to a liquid state of the materials that are expelled from the cellule, and absorbed into the torrent of the circulation;—or from an excessive tendency of the organised matter to become liquefied; or from a morbid predominance of the excretory process attracting all the organic matter within its reach." Having laid down these principles, Dr. Caustall proceeds to examine the internal and external influence, which are apt to induce the state of atrophy in any part.—*Ibid, from Handworterbuch der Physiologie.*

